# Innovations in Peripheral Nerve Regeneration

**DOI:** 10.3390/bioengineering11050444

**Published:** 2024-04-30

**Authors:** Ting Chak Lam, Yiu Yan Leung

**Affiliations:** Division of Oral and Maxillofacial Surgery, Faculty of Dentistry, The University of Hong Kong, Hong Kong, China; u3507413@connect.hku.hk

**Keywords:** peripheral nerve injury, nerve regeneration, nerve repair, nerve guidance conduits, pharmacotherapy

## Abstract

The field of peripheral nerve regeneration is a dynamic and rapidly evolving area of research that continues to captivate the attention of neuroscientists worldwide. The quest for effective treatments and therapies to enhance the healing of peripheral nerves has gained significant momentum in recent years, as evidenced by the substantial increase in publications dedicated to this field. This surge in interest reflects the growing recognition of the importance of peripheral nerve recovery and the urgent need to develop innovative strategies to address nerve injuries. In this context, this article aims to contribute to the existing knowledge by providing a comprehensive review that encompasses both biomaterial and clinical perspectives. By exploring the utilization of nerve guidance conduits and pharmacotherapy, this article seeks to shed light on the remarkable advancements made in the field of peripheral nerve regeneration. Nerve guidance conduits, which act as artificial channels to guide regenerating nerves, have shown promising results in facilitating nerve regrowth and functional recovery. Additionally, pharmacotherapy approaches have emerged as potential avenues for promoting nerve regeneration, with various therapeutic agents being investigated for their neuroprotective and regenerative properties. The pursuit of advancing the field of peripheral nerve regeneration necessitates persistent investment in research and development. Continued exploration of innovative treatments, coupled with a deeper understanding of the intricate processes involved in nerve regeneration, holds the promise of unlocking the complete potential of these groundbreaking interventions. By fostering collaboration among scientists, clinicians, and industry partners, we can accelerate progress in this field, bringing us closer to the realization of transformative therapies that restore function and quality of life for individuals affected by peripheral nerve injuries.

## 1. Introduction

Peripheral nerve injury pertains to the affliction or impairment of nerves located outside the confines of the brain and spinal cord, which play a crucial role in transmitting signals to and from different parts of the body. The prevalence of peripheral nerve injuries varies widely, ranging from 0.13% to 5% [1,2]. These injuries can be classified according to Sunderland’s classification, which is based on histological features. Grade I indicates neurapraxia, characterized by focal segmental demyelination. Grades II–IV represent axonotmesis, involving damaged axons with or without concurrent injury to the endoneurium, perineurium, and epineurium. Grade V signifies neurotmesis, indicating complete nerve transection [3]. Peripheral nerve injuries could give rise to a diverse range of symptoms, encompassing mild dysesthesia, paresthesia, allodynia, or pain. These symptoms significantly impede functional coordination and considerably disrupt daily lives of affected patients. Development of depressive symptoms are found related to individuals with permanent trigeminal neurosensory deficit, especially in the elderly [4,5,6].

Despite the inherent regenerative ability of peripheral nerves following injury, the process of healing and regeneration remains unpredictable and dependent on the nature and severity of the injury. As a result, achieving complete sensory or functional recovery after peripheral nerve injury remains a challenging outcome. Consequently, researchers have devoted extensive efforts to unravel the mechanisms underlying peripheral nerve injury, healing, and regeneration. The quest for potential treatments or therapies to enhance peripheral nerve healing continues to captivate the minds of neuroscientists, leading to a surge in publications and a growing interest in this field.

This article aims to contribute to the existing knowledge by providing a comprehensive review from both a biomaterial and clinical standpoint. Specifically, it will delve into the realm of nerve guidance conduits and pharmacotherapy, shedding light on the recent advancements made in peripheral nerve healing. Nerve guidance conduits offer a promising approach to guide and support nerve healing, while pharmacotherapy explores the potential of medications and vitamin supplementation to alleviate symptoms from neurosensory deficit and enhance functional recovery based on their possible neuroprotective properties that promote the repair and regenerative process. By reviewing these advancements, this article aims to contribute to the existing knowledge and inspire further research in the pursuit of effective treatments for peripheral nerve injuries.

## 2. Peripheral Nerve Capacity of Self-Regeneration after Injury

The potential of self-regeneration of the peripheral nerve is attributed to the preservation of neuronal cell vitality. Injuries to the axons of peripheral nerves do not lead to insult on the neural cell bodies or ganglions that are usually protected by skeleton or bony structures, and loss of neuronal soma is usually avoided. Unlike oligodendrocytes in the central nervous system, Schwann cells facilitate regeneration by orchestrating cellular and biochemical events throughout the post injury period in the peripheral nervous system. Immediately after nerve injury, a process known as Wallerian degeneration, as proposed by Dr Waller in 1850, occurs and persists for several weeks [7]. This process involves the degeneration of axons and myelin, followed by organized phagocytosis of cellular debris. Wallerian degeneration starts within hours after nerve injury. The distal axon and myelin disintegrate while the proximal axon and neuronal cell body remain intact. Schwann cells in the distal nerve stump become activated and proliferate, followed by secretion of various neurotrophic factors and cytokines that stimulate axonal regeneration. Subsequently, the regeneration process that is primarily mediated by Schwann cells ensues, allowing possible reinnervation of the end organ and the restoration of sensory and motor functions.

### 2.1. Role of Schwann Cells in Healing Peripheral Nerve Injury

From a molecular perspective, Schwann cells play a crucial role in the regenerative process following nerve injury. These cells possess a remarkable capacity for plasticity and can adaptively respond to the injury [8]. One of the key mechanisms by which Schwann cells contribute to nerve regeneration is through myelin reversal. This process involves the downregulation of specific myelin-inducing transcription factors, such as Egr2 (Krox20), myelin basic protein, and myelin-associated glycoprotein (MAG) [9]. In addition to myelin reversal, Schwann cells undergo further reprogramming and acquire a novel phenotype known as “repair supportive Schwann cells”. This phenotype is characterized by an upregulation of neurotrophic factors that promote the survival of remaining axons [10]. These neurotrophic factors create a supportive environment for axonal growth and regeneration. Furthermore, repair supportive Schwann cells stimulate inflammatory responses by attracting and recruiting macrophages to the site of injury. Macrophages, in turn, aid in myelin breakdown through the upregulation of cytokines like TNFα, leukemia inhibitory factor (LIF), interleukin-1α (IL-1α), and IL-6 [11]. These cytokines not only facilitate myelin clearance but also directly promote axonal regeneration. The role of macrophages extends beyond myelin breakdown and the promotion of inflammatory responses. They also contribute to sustaining the inflammatory cytokine milieu, support nerve vascularization [12], and assist in the clearance of debris and cellular waste products, including myelin remnants [13]. The coordination between Schwann cells and macrophages is crucial for creating an environment conducive to nerve regeneration. Of paramount importance, repairing Schwann cells provides guidance for regenerating axons. Within the endoneurial sheaths, these cells proliferate and undergo a transformation from a flattened myelin-type Schwann cell morphology to elongated longitudinal cells densely packed in the structure known as the bands of Bungner. These bands of Bungner are formed within the basal lamina tubes [14]. Regenerating axons, guided by the bands of Bungner, grow at an approximate rate of 0.25 mm per day until they reach the distal endoneurium, where reinnervation can occur. In conditions where the nerve is well vascularized, such as in nerve grafts, the growth rate of regenerating axons can be significantly increased to approximately 3–4 mm per day [15].

Understanding the molecular mechanisms underlying Schwann cell plasticity and their interactions with other cellular components, such as macrophages, is crucial for developing therapeutic strategies to enhance nerve regeneration. Manipulating the expression of specific factors involved in myelin reversal and the transition to a repair-supportive phenotype, as well as modulating the inflammatory response and promoting axonal guidance, may hold promise for promoting more robust and efficient nerve regeneration. Additionally, further research into the interactions between Schwann cells, macrophages, and other cellular and molecular components will uncover new insights into the complex processes underlying nerve regeneration and potentially lead to innovative therapeutic interventions for nerve injuries.

### 2.2. Limitations in Self-Regeneration

The regenerative potential of Schwann cells, although remarkable, can be hindered by various factors, including tissue aging and chronic denervation of the distal stumps. Chronic denervation refers to a prolonged period of disconnection between the nerve fibers and their target tissues, leading to a decline in the number of repairing Schwann cells. This decrease occurs due to a decrease in the differentiation of Schwann cells into new repair-supportive Schwann cells and ongoing cell death [16]. From a biochemical perspective, the transcription factor c-Jun plays a crucial role in maintaining the repairing process. Studies conducted on c-Jun-knockout mice have revealed that the absence of c-Jun results in suppressed myelinophagy (the degradation of myelin debris) and delayed downregulation of myelin-related molecules [17]. In vitro studies have further demonstrated that Schwann cells lacking c-Jun fail to adopt the typical bipolar columnar morphology associated with the repair-supportive phenotype. Instead, these cells remain flattened and exhibit disorganized regeneration tracks of the bands of Bungner, which are essential for guiding regenerating axons [18]. The c-Jun transcription factor plays a vital role in orchestrating the reprogramming of Schwann cells, and maintaining adequate levels of c-Jun is crucial for sustaining the quantity and quality of repair-supportive Schwann cells necessary for mediating the entire repair and regeneration process [19]. Therefore, it is not uncommon in daily clinical practice to encounter situations where the innate regenerative properties of injured peripheral nerves are insufficient, resulting in prolonged signs and symptoms. 

To overcome these limitations and enhance nerve regeneration, ongoing research aims to develop strategies that target the molecular mechanisms involved in Schwann cell plasticity and the maintenance of repair-supportive phenotypes. Understanding the intricate signaling pathways and transcriptional networks that regulate Schwann cell behavior can potentially lead to the identification of therapeutic targets. Modulating the expression of key transcription factors, such as c-Jun, may offer new avenues for promoting the differentiation and function of repair-supportive Schwann cells. Furthermore, promoting a conducive environment for nerve regeneration is equally important. Approaches that address tissue aging, such as the use of regenerative biomaterials or stem cell-based therapies, hold promise for rejuvenating the regenerative potential of Schwann cells and optimizing the overall regenerative milieu. Combining these strategies with techniques that enhance neurotrophic support, axonal guidance, and vascularization can further augment the regenerative capacity of injured peripheral nerves.

## 3. Contemporary Treatment to Peripheral Nerve Injury

**Figure 1:**
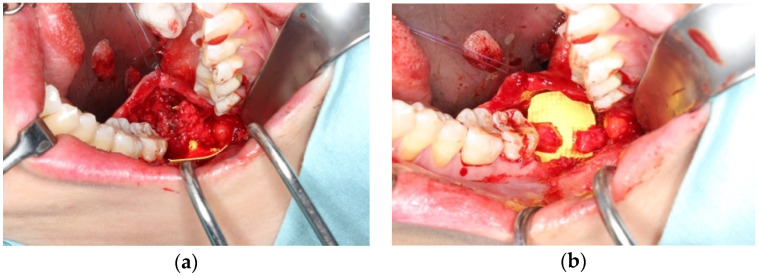
Lingual nerve neurotmesis after lower third molar surgery; (**a**) traumatic neuroma composed of scar tissue at iatrogenic lingual nerve injury site; (**b**) nerve gap after excision of the neuroma, exposing healthy nerve fascicles.

Surgical intervention is typically essential when the axonal injury is severe enough to surpass the innate regenerative capabilities of the body, resulting in persistent signs and symptoms. In Sunderland Class I–III injury, which might commonly include crushing and chemical cauterization, monitoring by neurosensory testing such as von Frey fibers and two-point discrimination could be adopted. When symptoms persist or exacerbate, or in Sunderland Grade IV or V injury, micro-neurosurgery that aims for reconnection and regeneration of axons to allow intended nerve impulses to occur would be considered. External neurolysis, as defined by Seddon [20], is a commonly employed procedure aimed at releasing the nerve from scar tissue to facilitate regeneration (Figure 1a,b). The main goal of the procedure is to relieve external compression on the nerve by freeing fibrotic and scarred tissue surrounding it. Once the nerve is released from the surrounding scar tissue, healthy neural tissue is exposed that allows recovery. While rarely performed as an independent procedure, it is always carried out as the initial step in all nerve lesions to expose healthy vascularized nervous tissue for further reconstruction. Following external neurolysis, additional procedures such as neurorrhaphy and nerve grafting can be performed, depending on the extent and severity of nerve injury.

**Figure 2:**
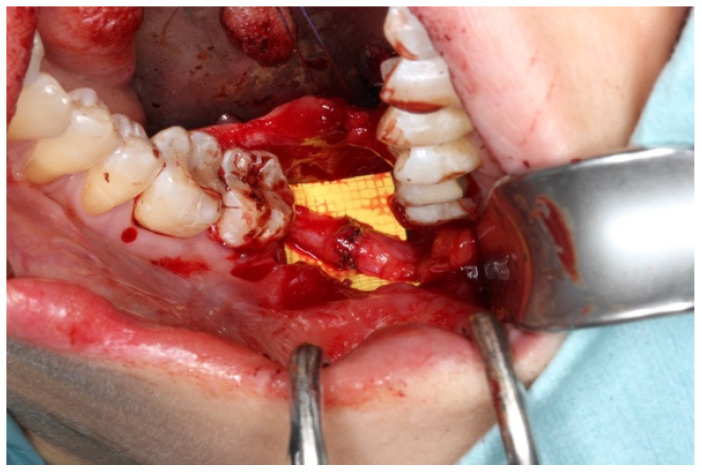
Tensionless neurorrhaphy performed with non-resorbable sutures allows best healing outcome from the surgical repair.

When a gap or discontinuity exists in a nerve, the preferred method of repair is neurorrhaphy through epineural suturing if the proximal and distal stumps can be brought together without tension. It is important to note that the ideal gap size for successful neurorrhaphy is generally considered to be smaller than 10 mm [21]. This approach aims to restore the continuity of the nerve and promote proper nerve regeneration (as depicted in Figure 1b). An illustrative example of a microsurgically anastomosed lingual nerve is shown in Figure 2.

In the context of lingual nerve repair, tensionless microsurgery has shown promising improvements in patient outcomes. A study conducted by Leung et al. demonstrated that 80% of patients experienced improvements in static light touch, while 70% of patients showed enhancements in two-point discrimination following lingual nerve repair [22]. Moreover, most patients reported improvements in taste sensation, indicating successful functional recovery. Additionally, 40% of patients reported a recovery in pain threshold, indicating a reduction in neuropathic pain. However, it is important to acknowledge that a small percentage (10%) of patients complained of a deterioration of sensation after surgical repair [22]. The precise reasons behind the deterioration of sensation after surgical repair are not yet fully understood, but several factors may contribute to this outcome. Firstly, the challenging clinical access in the oral cavity presents difficulties in achieving optimal surgical outcomes. The intricate anatomy and limited space can make the procedure technically demanding, potentially affecting the precision of the repair. Further, the unpredictable formation of fibrous scar tissue impedes axonal regeneration and contributes to the deterioration of sensation post-surgery. These scar tissues might create a physical barrier that hinders the growth of regenerating nerve fibers, preventing them from properly reestablishing connections.

An alternative approach that has garnered attention in the field of nerve reconstruction involves the use of fibrin glue. This innovative technique has been proposed as a viable option, showcasing comparable overall axonal regeneration, fiber alignment, and nerve conduction velocities when compared with traditional methods such as direct epineural suturing. Another notable advantage of fibrin glue is its ability to elicit a lesser granulomatous inflammatory response, which can contribute to improved patient outcomes and reduced complications. 

### 3.1. Nerve Grafting and Guidance Conduits

In situations where achieving tension-free neurorrhaphy is challenging, autografts are widely regarded as the gold standard for nerve reconstruction. These grafts are favored due to their minimal immunological reactions and their ability to provide an optimal regenerative microenvironment for nerve healing [23]. Among the various options available, the sural nerve, a sensory nerve extracted from the patient’s lower limb, is commonly utilized as the preferred donor source [24]. Its suitability stems from its accessibility and compatibility with the recipient site, facilitating successful nerve regeneration. Besides autologous nerve grafts, vein grafts are also commonly utilized. The use of saphenous vein or facial vein has been reported by Pogrel et al. [25] in repairing lingual nerve or inferior alveolar nerve defects with nerve gap ranges from 2 to 14 mm with considerable outcome. 

However, it is important to acknowledge that autografts are not without their drawbacks. One notable limitation is the potential for donor site morbidity, which can lead to post-operative complications and prolonged recovery periods. Additionally, the availability of grafting materials is inherently limited, which can pose challenges when multiple nerve repairs or extensive reconstructions are required. 

To overcome the limitations associated with autografts, artificial nerve guidance conduits are utilized to bridge nerve gaps. Bell et al. compiled a summary of FDA-approved commercially available nerve guidance conduits or wraps [26] that are composed of natural or synthetic polymers and allografts (Table 1). Most of these conduits adopt a basic tubular design to provide intraluminal guidance for nerve regeneration, with the exception of Avance^®^ (Axogen, Alachua, FL, USA), which utilizes nerve allografts to take advantage of the laminin-rich endoneurium. Generally, the maximum regeneration distance is limited to 20–25 mm [26], although products such as NeuraGen^®^ (Integra Life Science Corp, Princeton, NJ, USA) and NeuraMend^®^ (Stryker, Kalamazoo, MI, USA) offer options that are 30 mm and 50 mm in length, respectively. A multicenter study conducted by Bauback et al. [27], involving 624 nerve repairs, even demonstrated successful repairs of up to 70 mm with 82% meaningful recovery in terms of sensory, mixed, and motor nerve repairs using Avance^®^ allografts.

Despite the considerable progress made in the development of nerve guidance conduits, several limitations and challenges persist in their clinical application often resulting in less satisfactory healing outcomes compared with autografts. In the subsequent section, we delve into recent advancements in artificial nerve guidance conduits and explore potential solutions to overcome these limitations.

### 3.2. Application in Oral and Maxillofacial Surgery

Inferior alveolar (IAN) and lingual nerves (LN) are the two most encountered nerves in the field of oral and maxillofacial surgery. Ducic et al. [28] reported a study comprising 478 micro-neurosurgical reconstructions, with primary neurorrhaphy, allograft, and conduit. It was reported that, in situations where gap reconstructions were required, nerve allografts and autografts were found superior to conduits in both IAD and LN reconstructions in both sensory and functional recovery. However, it is interesting that this study reported no significant difference among primary repair, allografts, and autografts with regard to sensory and functional recovery. Therefore, it is believed that neurorrhaphy is still the most adopted treatment modality, especially when tensionless repair can be carried out.

### 3.3. Pharmacotherapy in Treating Nerve Injury

In addition to surgical intervention, various medications and pharmaceutical agents are commonly prescribed to alleviate and manage symptoms, particularly neuropathic pain resulting from nerve injury. These medications play a crucial role in improving the quality of life for patients by reducing pain and discomfort. One commonly prescribed medication for neuropathic pain is gabapentin, which is typically administered at a dosage of 300 mg per day. However, it is important to note that variations in titration schemes exist among different regions and individual medical practitioners’ preferences. While gabapentin has proven effective in addressing neuropathic pain, there is a lack of widely used medications that have demonstrated promising results in improving other symptoms such as numbness or paraesthesia, which can significantly impact a patient’s daily functioning and overall well-being. 

However, an intriguing avenue of exploration lies in the utilization of vitamin B complex to potentially enhance symptoms associated with peripheral nerve injury. In recent years, an increasing number of studies have focused on investigating the effects of vitamin B complex supplementation on peripheral nerve injury. These studies aim to elucidate the underlying mechanisms and determine the optimal dosage and duration of treatment. For example, Baltrusch et al. conducted a study examining the effects of vitamin B complex on nerve regeneration in a rat model of sciatic nerve injury. Their findings revealed that vitamin B complex supplementation resulted in improved functional recovery, increased myelinated nerve fibers, and enhanced axonal diameter [29].

Despite these promising findings, the clinical outcomes of employing vitamin B complex as a standard treatment for peripheral nerve injury remain controversial. A Cochrane Review, which analyzed existing randomized controlled trials on the treatment of peripheral neuropathy with vitamin B complex, reported inconclusive results. This lack of consensus makes it difficult to determine the overall benefits or potential harm of vitamin B complex supplementation in peripheral nerve injury [30]. Altun et al. [31], in their research, reported variations in vitamin B12 levels during the progression of healing after peripheral nerve injury. They suggested that supplementation of these vitamins might be beneficial during the acute phase of nerve healing following injury. This observation raises the possibility that the timing of vitamin B complex supplementation may influence treatment outcomes. Patients who typically experience chronic neuropathic pain or paraesthesia following nerve injury may not receive the same benefits as those who receive supplementation during the acute phase of healing [31]. The conflicting results and uncertainties surrounding the use of vitamin B complex in peripheral nerve injury highlight the need for further research. Future studies should focus on larger sample sizes, well-designed clinical trials, and standardized protocols to evaluate the efficacy of vitamin B complex supplementation. Understanding the underlying mechanisms of action and identifying patient subgroups that may benefit the most from this treatment approach will be crucial in optimizing its use.

To enhance functional recovery following peripheral nerve repair and regeneration, there is growing interest in the use of other adjunctive pharmacotherapy to promote more robust healing outcomes. Steroids, including estrogen and progesterone, have been proposed as potential agents based on in vitro and in vivo evidence highlighting their neuroprotective properties and ability to upregulate myelination [32]. However, it is crucial to acknowledge that steroids carry significant side effects, such as an increased risk of breast cancer and deep vein thrombosis [33]. Therefore, their use must be carefully considered and evaluated on a case-by-case basis, weighing the potential benefits against the potential risks. Another pharmaceutical agent that has garnered considerable attention in the field of peripheral nerve injury is erythropoietin. Although primarily used for the treatment of anemia, erythropoietin has been extensively studied for its neuroprotective effects and has shown promising results [34,35,36]. Clinical trials investigating the therapeutic use of erythropoietin specifically for peripheral nerve trauma have been conducted, aiming to explore its potential benefits in promoting nerve regeneration and functional recovery [37]. However, it is important to approach erythropoietin therapy with caution, as it is a hematopoietic agent and systemic adverse events have been reported. Instances of coronary stent thrombosis and deep vein thrombosis have been documented, highlighting the need for careful monitoring and consideration of potential risks [38]. While the use of steroids and erythropoietin as adjunctive pharmacotherapy in peripheral nerve injury holds promise, further research is warranted to fully understand their application, benefits, and potential risks. Rigorous studies, including well-designed clinical trials, are necessary to establish the optimal dosage, timing, and duration of treatment, as well as to assess long-term outcomes and potential adverse effects. Additionally, exploring the mechanisms of action of these pharmacological agents in promoting nerve regeneration and functional recovery will contribute to a deeper understanding of their therapeutic potential.

**Figure 3:**
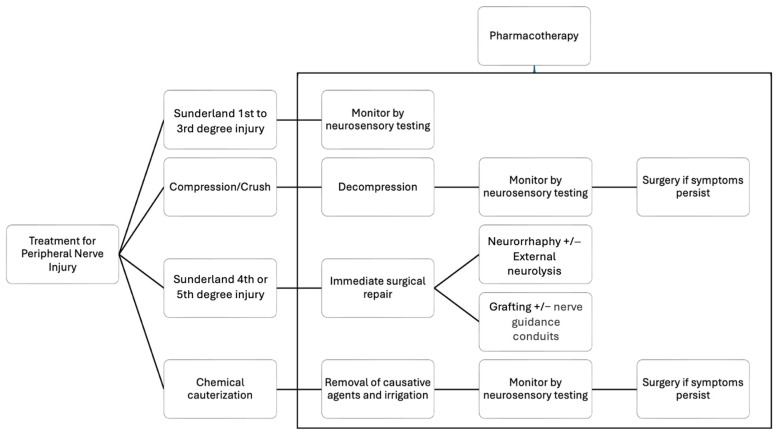
Diagram summarizing contemporary treatment for peripheral nerve injuries (modified from Miloro, M. Chapter 25. Microneurosurgery. In Peterson’s Principles of Oral and Maxillofacial Surgery, 4th ed.) [39].

Figure 3 summarizes the contemporary treatment for peripheral nerve injury from a clinical point of view, while pharmacotherapy might exert their effect at different time points, promoting nerve healing either to avoid surgical intervention or enhancing healing after micro-neurosurgery [39].

## 4. Nerve Guidance Conduit

Nerve guidance conduits have emerged as a highly promising and innovative solution to address the limitations associated with autologous nerve grafting or contemporary neurorrhaphy techniques [40]. Extensive studies have demonstrated that when nerve gaps exceed 3 mm, traditional tensionless neurorrhaphy techniques face significant challenges, thereby necessitating the utilization of nerve guidance conduits [41]. These conduits serve as a vital role as connectors, bridging the substantial defect between the proximal and distal ends of the injured nerve. Their primary goal is to facilitate Schwann cell proliferation, which plays a crucial role in guiding nerve regeneration. 

One of the primary objectives of nerve guidance conduits is to provide a microenvironment that prevents the infiltration of fibrous tissue while simultaneously promoting the organized outgrowth of axons. This is essential in minimizing disorganized sprouting and the formation of glial scars, which can impede proper nerve regeneration. In an ideal scenario, nerve guidance conduits should possess specific characteristics, including biodegradability, flexibility, permeability for the exchange of trophic factors and metabolic waste during the healing process, mechanical support, and biomimetic properties [42]. Initially, non-porous and non-resorbable silicon was employed as a primitive conduit material in the 1980s [43]. However, subsequent research endeavors delved into exploring the potential of various natural and synthetic materials, leading to the development of diverse conduit designs. These designs encompass porous structures, grooves or aligned nanofibers on the inner surface, composite bi-layered structures, and the incorporation of growth factors to enhance regenerative process.

In recent years, significant advancements have been achieved in the bioengineering modification of materials and designs utilized in nerve guidance conduits. Among the wide array of natural polymers of interest, collagen and chitosan have emerged as the most popular choices for serving as the main structural components. While natural polymers provide a favorable environment for regeneration, synthetic polymers are often combined simultaneously in composite conduit designs to enhance mechanical and structural stability. The manufacturing methods for nerve guidance conduits have also witnessed rapid changes. The manufacturing methods for nerve guidance conduits have witnessed rapid changes. In addition to commonly used techniques such as electrospinning, recent studies have highlighted alternative fabrication methods, including freeze drying and 3D printing [44]. Notably, the implementation of 3D printing technology has allowed for development of patient-specific nerve guidance conduits that can be customized to match the individualized morphology of the injured nerve [45]. Furthermore, Krishna et al. reported utilization of artificial intelligence for improving properties of bioengineering conduit scaffold properties in 3D printing patient-specific nerve guidance conduits [46]. The advancement of machine learning holds great potential for revolutionizing the development of nerve guidance conduits, particularly in addressing the clinical challenges associated with severe nerve injuries characterized by complex morphologies and significant discontinuities.

In addition to addressing structural aspects, the integration of neurotrophic factors has emerged as a crucial element in enhancing nerve regeneration. Ongoing research in the field of nerve guidance conduits is focused on exploring innovative methods to incorporate growth factors into different conduit materials, aiming to optimize their regenerative effects. By conjugating these growth factors with conduit materials, nerve guidance conduits serve as platforms for the sustained release of bioactive concentrations throughout the entire regenerative process.

In recent studies, promising outcomes have been observed through the conjugation of specific growth factors with various conduit materials. For instance, neuregulin-1 has been successfully conjugated with flexible phosphorene hydrogel conduits, resulting in positive regenerative effects [47]. Similarly, the incorporation of neurotrophin-3 with silk fibroin conduits has shown favorable outcomes in promoting nerve regeneration [48]. Basic fibroblast growth factor (bFGF) and nerve growth factor (NGF) have been conjugated with heparin and polycaprolactone/gelatin conduits, demonstrating encouraging results as well [49]. Furthermore, a study by Qi et al. [50] took a unique approach by incorporating platelet-rich plasma into gelatin and alginate hydrogel, creating a model for the sustained release of vascular endothelial growth factor (VEGF) and platelet-derived growth factor-BB (PDGF). This innovative method exhibited significant increases in Schwann cell migration in vitro and promoted axonal regeneration in vivo. These advancements highlight the potential of growth factor incorporation in nerve guidance conduits to enhance the regenerative process. By providing a controlled and sustained release of growth factors, these conduits create an optimal environment for nerve regeneration. The successful conjugation of specific growth factors with various conduit materials opens new avenues for tailored treatment approaches in nerve regeneration.

Moreover, the utilization of complexes such as extracellular matrix and exosomes have garnered increasing popularity in research due to their potential to contain multiple factors that favor regeneration. Decellularized extracellular matrix [51,52,53,54] is believed to provide an optimal microenvironment for cell growth and viability, containing a rich network of glycosaminoglycans and neurotrophic growth factors that promote Schwann cell proliferation and facilitate nerve regeneration after decellularization [55]. Hibbitts et al. [56] have even explored the specific ratios of ECM components, such as fibronectin and laminin, to optimize axonal regeneration. Exosomes contain extracellular vesicles generated by cells that contain proteins, nucleic acids, and other metabolites. While not widely recognized for clinical treatment by authorities such as the FDA, exosomes have gained popularity in in vivo studies in recent years. Exosomes derived from Schwann cells [57] and stem cells from various tissues, including bone marrow [58], human umbilical cord mesenchyme [59], adipose tissue [60,61], and human endometrium [62] have been extensively studied in sciatic nerve repair. The results have shown significantly improved functional recovery and reduced muscle atrophy comparable to the gold standard of autografts. These emerging approaches utilizing complexes such as decellularized ECM and exosomes hold great promise for enhancing nerve regeneration and functional recovery. 

However, further research is still necessary to fully understand the optimal combinations of growth factors and exosomes with conduit materials, as well as the ideal release kinetics for effective nerve regeneration. Research regarding these perspectives remains largely diversified. Comprehensive studies are needed to assess the long-term effects, safety, and scalability of these growth factor conduit systems. By addressing these aspects, researchers can continue to refine and optimize the use of growth factors and exosomes in nerve guidance conduits, ultimately leading to more successful and clinically applicable regenerative therapies for peripheral nerve injuries.

## 5. Pharmacotherapy

In a recent publication by Souza et al. [63], a comprehensive and meticulous review was conducted to investigate the efficacy and potential applications of a wide range of pharmacotherapeutic agents in various therapeutic contexts. The study delved into both herbal medicines, such as gingko biloba and curcumin, and conventional medicine like dexamethasone. By analyzing existing research, the authors aimed to provide valuable insights into the therapeutic effects of these agents. The review highlighted the potential benefits of herbal medicines in promoting neuroregeneration. Gingko biloba, known for its antioxidant and anti-inflammatory properties, has shown promise in improving cognitive function and reducing neuronal damage in neurodegenerative disorders [61]. Curcumin, a compound found in turmeric, has also exhibited neuroprotective effects and anti-inflammatory properties, showing potential in mitigating neurodegenerative processes [63]. The inclusion of these herbal medicines in the review broadens the scope of potential therapeutic interventions and emphasizes the importance of considering alternative approaches to conventional medicine.

Additionally, Manto et al. [64] proposed a novel approach for systemic administration of 4-aminopyridine (4-AP) using a thermosensitive polymer called poly(lactide-co-glycolide)−b-poly(ethylene glycol)−b-poly(lactide-co-glycolide) (PLGA−PEG−PLGA). Their research focused on the enhanced delivery of 4-AP, a potassium channel blocker known to improve axonal conduction and functional recovery in neurological disorders. The use of this thermosensitive polymer allowed for controlled and sustained release of 4-AP, leading to improved motor and sensory functional recovery in vivo and increased expression of neuroregeneration markers in vitro [64]. These findings hold promise for the development of targeted and efficient pharmacotherapeutic interventions in the field of neuroregeneration.

Among the various therapeutic agents explored, erythropoietin has emerged as a subject of extensive investigation due to its remarkable neuroprotective properties. Despite recognized side effects and challenges associated with its clinical application, researchers have continued to delve into the potential benefits of this glycoprotein hormone. Notably, Talukder et al. conducted groundbreaking research utilizing genetically modified knock-out mice models to elucidate the indispensable role of Schwann cell-specific erythropoietin receptors (EpoRs) in neuroregeneration [65]. Their findings provide compelling evidence supporting the neuroregenerative properties of erythropoietin and underscore the need for further exploration of its therapeutic potential. Recent studies have also revealed intriguing possibilities for synergistic effects between erythropoietin and other therapeutic agents. For instance, simultaneous administration of dexamethasone and erythropoietin has shown promising results, suggesting potential complementary actions that enhance neuroprotective outcomes [66,67]. 

It is worth noting that derivatives of erythropoietin, such as carbamylated erythropoietin (CEPO), have garnered increasing attention due to their ability to retain the neuroprotective properties of erythropoietin while circumventing its hematological effects. Leist et al. suggested the neuroprotective effects of carbamylated erythropoietin in various animal models with neuropathy due to diseases such as ischemic stroke, sciatic nerve compression, spinal cord depression, and peripheral diabetic neuropathy [68,69]. Importantly, these studies have demonstrated that the long-term administration of high doses of CEPO does not elicit erythropoietic effects, thereby minimizing concerns associated with hematological complications. Further research, as suggested by Chen et al., is required to deepen our understanding of the biochemical mechanisms underlying the neuroprotective properties of CEPO [68]. Their findings, along with other recent studies, strongly support the potential of CEPO as a promising drug candidate for central nervous system (CNS) diseases, including but not limited to traumatic brain injury, periventricular leukomalacia, and Parkinson’s disease. However, the application of CEPO in peripheral nerve injuries remains an area that necessitates extensive investigation and future research efforts.

The recent publications and studies discussed in this paragraph significantly contribute to our understanding of the therapeutic potential of various pharmacotherapeutic agents, with a particular focus on erythropoietin and its derivatives. These investigations shed light on the neuroprotective properties of these agents and pave the way for potential advancements in neuroregeneration research. While the reviewed pharmacotherapeutic agents demonstrated positive outcomes in terms of functional recovery and axonal regeneration, it is important to acknowledge the complexity and diversity of these investigations. The field of pharmacodynamics is multifaceted, and there are various factors that can influence the outcomes of these agents. Further research is necessary to unravel the underlying mechanisms and optimize the therapeutic potential of these agents. Future studies should focus on elucidating the specific molecular pathways and cellular mechanisms through which these pharmacotherapeutic agents exert their effects on neuroregeneration. Understanding these mechanisms will facilitate the development of more targeted and precise interventions. Additionally, investigating the optimal dosage, timing, and duration of treatment for each agent will contribute to optimizing their therapeutic efficacy. Moreover, it is essential to conduct rigorous clinical trials to evaluate the safety and long-term effects of these pharmacotherapeutic interventions. Adverse effects, drug interactions, and individual variations in response should be carefully monitored and considered.

## 6. Conclusions

To reiterate, the recent advancements in research on nerve guidance conduits and pharmacotherapy have shown great promise in the treatment of peripheral nerve injuries. These developments have significantly contributed to the field by addressing the challenges associated with nerve regeneration. However, the biomaterials for nerve guidance conduits and pharmaceutical agents under research remain diverse. Despite being seemingly promising, the majority of the targeted research still lack definitive results, requiring further research and in-depth investigations to explore their full potential. More in vivo studies and even clinical research on these biomaterials or pharmaceutical agents shall be carried out. These studies will help establish the treatment’s safety, efficacy, and optimal administration protocols in the context of peripheral nerve injury. Rigorous investigation and scientific inquiry are vital to ensure that these innovative treatments can be utilized effectively and safely in clinical settings. As the field of nerve repair and regeneration continues to evolve, research into novel biomaterials or agents such as exosomes, growth factors, and derivatives of erythropoietin may emerge as key therapeutic game changers in the management of peripheral nerve injury. Meaningful relief and improved functional outcomes may become a reality for patients affected by peripheral nerve injuries.

## Figures and Tables

**Table 1:** List of FDA-approved commercially available nerve guidance conduits or wraps that can be used to bridge nerve gaps.

Brand Name	Company	Material
NeuraGen^®^, Neurawrap^TM^	Integra Life Science Corp, Princeton, NJ, USA	Type I collagen
NeuroMatrix^TM^, Neuroflex^TM^, NeuroMend^TM^	Stryker, Kalamazoo, MI, USA	Type I collagen
Neurolac^®^	Polyganics, Groningen, The Netherlands	PDLLA/CL
Neurotube^®^	Synovis, Birmingham, AL, USA	PGA
Salubridge^TM^, Salutunnel^TM^	Salumedica, Atlanta, GA, USA	Polyvinyl alcohol hydrogel
Surgrisis^®^, Axoguard^TM^	AxoGenInc, Alachua, FL, USA	Porcrine small intestinal submucosa
Avance^®^	AxogenInc, Alachua, FL, USA	Decellularized human nerve allograft
Reaxon^®^Direct	Kerimedical, Genève, Switzerland	Chitosan
RevolNerv^®^	Orthomed, Saint-Jeannet, France	Collagen Type I and III from porcine skin
